# The Occurrence and Relationship of Postoperative Seizure and de novo Epilepsy after Craniotomy Surgery: A Retrospective Single-Center Cohort Study

**DOI:** 10.3389/fsurg.2022.881874

**Published:** 2022-04-19

**Authors:** Sayaka Horiuchi, Kohei Kanaya, Tetsuyoshi Horiuchi

**Affiliations:** ^1^ Medical student, Shinshu University School of Medicine, Matsumoto, Nagano, Japan; ^2^ Department of Neurosurgery, Shinshu University School of Medicine, Matsumoto, Nagano, Japan

**Keywords:** seizure, epilepsy, postoperative, craniotomy, neurosurgery, antiseizure medication (ASM)

## Abstract

**Objective:**

Postoperative seizures and epilepsy are common complications of craniotomy. In this study, we aimed to investigate the characteristics of seizures and epilepsy after craniotomy.

**Methods:**

A total of 293 consecutive craniotomy surgeries were analyzed. Infratentorial surgeries, epilepsy surgeries, surgeries using the same approach conducted for the same patients, and the cases with incomplete clinical data were excluded. A total of 211 surgeries were included in this study. We evaluated the following clinical characteristics in all patients: sex, age, preoperative epilepsy, use of preoperative antiseizure medication (ASM), indication for operation, early postoperative seizure (EPS), delayed postoperative seizure (DPS), and postoperative de novo epilepsy. The day of onset of EPSs was defined as within 7 days post-surgery, and the day of onset of DPSs was defined as later than 7 days and less than 60 days post-surgery.

**Results:**

Twenty-eight patients were previously diagnosed with epilepsy. Nine patients had EPSs (4.3%), and 10 patients had DPSs (4.7%). Seven cases of EPSs and six cases of DPSs were observed in 183 patients without previous epilepsy (3.8% and 3.3%, respectively). Three of the seven patients with EPSs (42.9%) and all six patients with DPSs (100%) developed de novo epilepsy. Postoperative de novo epilepsy was observed in 9 (4.9%) of the 183 patients without epilepsy. EPSs and DPSs were significant risk factors for epilepsy (*p* < 0.01). The odds ratios of EPSs and DPSs for the development of epilepsy were 12.71 (95% confidence interval [CI]: 3.94–112.80; *p* < 0.01) and 22.88 (95% CI: 5.38–55.72; *p* < 0.01), respectively. ASM was administered prophylactically to 51 patients. The prophylactic use of ASMs did not prevent EPSs or postoperative de novo epilepsy.

**Conclusion:**

EPSs and DPSs occurred in 4.3% and 4.7% of the patients, respectively, after craniotomy. Postoperative de novo epilepsy occurred in 4.9% of patients. This study revealed that EPSs and DPSs were risk factors for de novo epilepsy. Previous epilepsy was not a significant risk factor for EPSs. The prophylactic use of ASMs did not prevent EPSs or de novo epilepsy.

## Introduction

Postoperative seizures and epilepsy are common complications of craniotomy surgery. Postoperative seizures were considered as early postoperative seizures (EPS) and delayed postoperative seizures (DPS). Previous reports showed that postcraniotomy seizures occurred in 11.9% of patients who underwent decompressive craniectomy (1). EPS was observed in 6.1% of patients undergoing supratentorial tumor surgery (2), and 17% of patients who underwent supratentorial surgery developed postoperative epilepsy (3). Another report showed that 43% of patients who underwent meningioma surgery experienced DPS (4).

Postoperative seizures and epilepsy can differ depending on the patient, disease, pathology, type of surgery, and previous history (5). Although some reports showed the risk of epilepsy following stroke (6, 7), meningioma surgery (8), and craniotomy (9), the relationship between EPS, DPS, and de novo epilepsy after craniotomy is not well known. The purpose of this study is to investigate the occurrence and relationship of EPS, DPS, and de novo epilepsy after various neurosurgical treatments. Furthermore, this study also aimed to evaluate the effectiveness of prophylactic use of antiseizure medication (ASM). Our research can provide unique contributions to how to treat postoperative seizures and when to diagnose de novo epilepsy after craniotomy surgeries.

## Methods

### Patient Selection

Data were retrospectively collected from 293 consecutive craniotomy surgeries performed at the Neurosurgical Department of Shinshu University Hospital from January 2017 to May 2021. Infratentorial surgeries, epilepsy surgeries, surgeries using the same approach conducted for the same patients, and the cases with incomplete clinical data were excluded. A total of 211 surgeries were included in this study. The average observational period was 18.1 months. We evaluated the following clinical characteristics in all patients: sex, age, preoperative epilepsy, use of preoperative antiseizure medication (ASM), indication for operation, EPS, DPS, and de novo epilepsy. The day of onset of EPSs was defined as within 7 days post-surgery, and the day of onset of DPSs was defined as later than 7 days and less than 60 days post-surgery. Epilepsy was defined as the presence of any of the following conditions: (1) at least two unprovoked seizures occurring >24 h apart; (2) one unprovoked seizure and a probability of further seizures similar to the general recurrence risk after two unprovoked seizures, occurring over the next 10 years (10). De novo epilepsy was defined as postoperative newly diagnosed epilepsy in the observational period. This study was approved by the Ethics Committee of Shinshu University School of Medicine (IRB number 5391).

### Statistical Analysis

Data are presented as mean ± standard deviation. Fisher’s exact test was used to assess the relationship between EPS and DPS and epilepsy, and EPS and de novo epilepsy and ASM use. Univariate regression analysis was used to assess the strength of the association between EPSs and DPSs and postoperative de novo epilepsy. Statistical significance was set at *p* < 0.05. SPSS (version 27; SPSS, Inc., Chicago, IL, USA) was used for statistical analysis.

## Results

### Patient Characteristics

The patient characteristics are presented in Table 1. The study included 97 males and 114 females. The average age was 56.1 ± 18.4 (range 10–97) years. Twenty-eight patients were previously diagnosed with epilepsy. Nine patients had EPSs (4.3%), and ten patients had DPSs (4.7%). Among the 183 patients without epilepsy, seven patients had EPSs, and six patients had DPSs (3.8% and 3.3%, respectively). Among the 28 patients with epilepsy, two patients had EPSs, and four patients had DPSs (7.1% and 14.3%, respectively). Postoperative de novo epilepsy was observed in nine of the 183 patients without previous epilepsy (4.9%).

**Table 1:** Clinical characteristics.

Characteristics	
Number (male/female)	211 (97/114)
Age average (range)	56.1 ± 18.4 (10–97)
Patients with epilepsy	28
EPS (wo. epilepsy/de novo epilepsy)	9 (7/3)
DPS (wo. epilepsy/de novo epilepsy)	10 (6/6)
de novo epilepsy (total)	9

*EPS, early postoperative seizure; DPS, delayed postoperative seizure; wo, without.*

### Disease Characteristics, EPSs, DPSs, and de novo Epilepsy

The patient characteristics are presented in Table 2. Twelve patients of 41 gliomas (29.3%), five patients of 31 meningiomas (16.1%), two patients of 14 metastatic brain tumors (14.3%), one patient of 40 unruptured aneurysms (2.5%), and two patients of 12 moyamoya diseases (16.7%) had focal epilepsy preoperatively. Of the nine patients with EPSs, four cases of glioma, three cases of unruptured aneurysms, one case of moyamoya disease, and one case of intracranial hemorrhage (ICH) were observed. Among the patients with epilepsy, two had EPSs. Among the patients without epilepsy, seven had EPSs. EPSs only occurred in four cases, and three cases of EPSs developed into postoperative de novo epilepsy. Four cases of DPSs were observed in patients with epilepsy. Six cases had DPSs among the patients without epilepsy, and all six patients with DPSs with no history of epilepsy developed postoperative de novo epilepsy. The average diagnosis period from craniotomy to the onset of de novo epilepsy was 22.3 (range 9–52) postoperative days.

**Table 2:** Clinical characteristics of epilepsy, EPS, DPS and de novo epilepsy.

Diseases	*n*	w. epilepsy, *n* (%)	EPS (*n* = 9)			DPS (*n* = 10)			de novo epilepsy, *n* (total) (%)
			w. epilepsy	wo. Epilepsy		w. epilepsy	wo. epilepsy		
				EPS only	de novo epilepsy		DPS only	de novo epilepsy	
glioma	41	12 (29.3%)	1	2	1	4	0	2	3 (10.3%)
meningioma	31	5 (16.1%)	0	0	0	0	0	3	3 (11.5%)
meta	14	2 (14.3%)	0	0	0	0	0	0	0 (0.0%)
unrup. An	40	1 (2.5%)	1	1	1	0	0	0	1 (2.6%)
rup. An	15	0 (0%)	0	0	0	0	0	0	0 (0%)
moyamoya	12	2 (16.7%)	0	0	1	0	0	0	1 (10.0%)
ICH	10	0 (0%)	0	1	0	0	0	1	1 (10.0%)
others	48	6 (12.5%)	0	0	0	0	0	0	0 (0%)
Total	211	28 (13.3%)	2	4	3	4	0	6	9 (4.9%)

*EPS, early postoperative seizure; DPS, delayed postoperative seizure; w, with; wo, without; meta, metastatic brain tumor; unrup. An, unruptured aneurysm; rup. An, ruptured aneurysm; moyamoya, moyamoya disease; ICH, intracranial hemorrhage.*

Three of the 29 gliomas (10.3%), three of the 26 meningiomas (11.5%), one of the 39 unruptured aneurysms (2.6%), one of the cases of 10 moyamoya diseases (10.0%), and one of the 10 ICHs (10.0%) developed into postoperative de novo epilepsy. In total, nine of the 183 patients who underwent craniotomy surgeries (4.9%) developed postoperative de novo epilepsy.

### The Relationship of EPSs, DPSs, and Previously Diagnosed Epilepsy

There was no significant difference between EPSs and the presence of preoperative epilepsy (*p* = 0.34); however, there was a significant difference between DPSs and the presence of preoperative epilepsy (*p* = 0.029) (Table 3). No significant relationship was observed between EPSs and the presence of preoperative epilepsy.

**Table 3:** The relationship of EPS, DPS and previously diagnosed epilepsy.

		Previous epilepsy		*p* value
		Yes	No	
EPS	Yes	2	7
	No	26	176	0.34
DPS	Yes	4	6
	No	24	177	**0** **.** **029**

*EPS, early postoperative seizure; DPS, delayed postoperative seizure.*

*Boldface type indicates statistical significance (p* *< 0.05).*

### The Relationship Between EPSs, DPSs, and Postoperative de novo Epilepsy

Of the 183 patients without epilepsy, three of the seven patients with EPS (42.9%) and all six patients with DPS (100%) developed postoperative de novo epilepsy. EPSs and DPSs were significant risk factors for epilepsy (*p* < 0.01). The odds ratios of EPSs and DPSs for the development of epilepsy were 12.71 (95% confidence interval [CI]: 3.94–112.80; *p* < 0.01) and 22.88 (95% CI: 5.38–55.72; *p* < 0.01), respectively (Table 4).

**Table 4:** The relationship and univariate regression analysis of EPS, DPS and de novo epilepsy.

		de novo epilepsy			
		Yes	No	Odds ratio of de novo epilepsy	*p* value
EPS	Yes	3	4	12.71 (3.94–112.80)
	No	0	176	1 (ref)	**<0** **.** **01**
DPS	Yes	6	0	22.88 (5.38–55.72)
	No	0	177	1 (ref)	**<0**.**01**

*EPS, early postoperative seizure; DPS, delayed postoperative seizure; ref, reference.*

*Boldface type indicates statistical significance (p < 0.05).*

### The Relationship Between Prophylactic use of ASMs and EPSs and de novo Epilepsy

ASM was administered prophylactically to 51 patients. Interestingly, the prophylactic use of ASMs did not prevent EPS; however, ASM use was also a risk factor for EPSs (*p* = 0.039). Furthermore, the prophylactic use of ASMs did not prevent postoperative de novo epilepsy (*p* = 0.71) (Table 5).

**Table 5:** The relationship of EPS, de novo epilepsy and prophylactic ASM.

		prophylactic ASM		*p* value
		Yes	No	
EPS	Yes	5	4
	No	46	156	**0** **.** **039**
de novo epilepsy	Yes	3	6
	No	48	126	0.71

*ASM, antiseizure medication; EPS, early postoperative seizure.*

*Boldface type indicates statistical significance (p < 0.05).*

## Discussion

Our study revealed that EPS and DPS occurred in 4.3% and 4.7% after craniotomy surgery, respectively. Three of the seven patients with EPS (42.9%) and all six patients with DPS (100%) developed de novo epilepsy. Postoperative de novo epilepsy occurred in 4.9% of patients after craniotomies in our study. Furthermore, the prophylactic use of ASMs did not prevent EPSs or de novo epilepsy.

Although the incidence of de novo epilepsy after craniotomies can be influenced by the observational period, a recent report showed that the overall 6-month, 1-year, and 5-year postoperative cumulative risks of de novo epilepsy after craniotomy were 9.7% (95% CI: 9.1–10.3), 13.9% (95% CI: 13.2–14.6), and 20.4% (95% CI: 19.5–21.3), respectively (9).

Our study revealed that DPSs and EPSs could be risk factors for de novo epilepsy. On the other hand, Beghi reported that early seizures alone are not sufficient to diagnose epilepsy, as they are deemed to be provoked (11). Seizures are considered acute symptomatic if they occur within the first 7 days of cerebrovascular disease (12, 13). Our data showed that all DPSs cases developed de novo epilepsy; on the other hand, about half of EPSs cases developed de novo epilepsy. We speculated that although EPSs can be similar to provoked seizures, DPSs can be related to unprovoked seizures, which is necessary for defining epilepsy. Therefore, DPSs seem to be a prominent factor for postoperative de novo epilepsy. Several studies have reported that seizure recurrence is more common in patients with late seizures than in those with early seizures (14, 15). Furthermore, Doria reported that late seizure following stroke had a high risk (55%–93%) for the development of epilepsy, although early seizure had a low risk (29%–35%) (16), which is concordant to our data although the patient cohorts were different. Early seizure after stroke may be related to acute neuronal injury and subsequent glutamate-mediated excitotoxicity, ion channel dysfunction, and blood barrier disruption (17–20). Late seizures after stroke may be related to secondary gliotic scarring with associated changes in membrane properties, chronic inflammation, neurodegeneration, and altered synaptic plasticity, eventually leading to hyperexcitability and increased synchronization of neuronal activities (21, 22).

In general, the prediction of postoperative seizures and de novo epilepsy may be challenging, Galovic proposed the SeLECT score to predict the risk of late seizures after stroke. The model incorporated five items: severity of stroke, large-artery atherosclerotic aetiology, early seizures, cortical involvement, and territory of middle cerebral artery involvement (7). Wirsching also reported that the predictors of poor postoperative seizure control after meningioma resection included preoperative epilepsy, epileptiform potentials on postoperative EEG recordings, severe surgical complications including CNS infections, hydrocephalus, recraniotomy, and symptomatic intracranial hemorrhage, younger age, and tumor progression (8).

Our study showed that previous epilepsy was a risk factor for DPSs but not EPSs. Furthermore, the prophylactic use of ASMs did not prevent EPSs and de novo epilepsy. Controversially, the prophylactic usage of ASMs was a risk factor for EPSs. This result suggests that the use of ASMs was not effective in preventing EPSs, although the possible risk factors of EPS such as the location of the lesion, indication for operation, and preoperative epilepsy were concerned. Previous studies have found no significant difference in the development of late seizures between patients receiving and not receiving prophylactic ASMs, suggesting that ASMs do not prevent epileptogenesis (23–25). Prophylactic ASM in patients with newly diagnosed brain tumors is not recommended because it is not effective in preventing seizures (26), and prophylactic ASM is not recommended routinely because of drug-related side effects (27). Postoperative seizures occur most often in the first week to the first month after surgery for patients with and without tumors (28, 29). If patients are treated perioperatively, tapering and discontinuing ASMs after the first postoperative week are recommended (26). The prophylactic use of ASMs was not effective in preventing postoperative seizures and epilepsy in our study. Therefore, ASM should be used based on the diagnosis of epilepsy. If ASMs are used prophylactically, they should not be used inappropriately for a longer time and should be discontinued in the early postoperative period.

The postoperative seizures were classified as either EPSs or DPSs. EPSs can be related to acute symptomatic seizures associated with craniotomy; however, DPSs can be comparable to unprovoked seizures, which may indicate seizures associated with epilepsy. Of seven DPS cases without previous epilepsy, four cases with focal motor seizure (FMS), one case with focal impaired awareness seizure (FIAS), and one case with focal to bilateral tonic-clonic seizure (FBTCS) were observed as DPSs based on the basic ILAE 2017 operational classification of seizure types (30).

The day of seizure onset after craniotomy is an important factor in diagnosing epilepsy. It can be hard to diagnose epilepsy if only one seizure occurs, especially during the early postoperative period. We consider these cases are preferred to be followed conservatively and carefully without administering ASMs. It is estimated that 20%–30% of cases are indeed misdiagnosed as epileptic seizures (31, 32). Perrig emphasized that patients should not be treated if there is uncertainty about the diagnosis of epilepsy, and “wait and see” for the next event can prevent the misdiagnosis of epilepsy (33).

This study has several limitations. First, this was a retrospective analysis of patients treated at a single institution with a relatively small sample size. Second, the follow-up duration varied among the patients. The incidence of delayed postoperative seizures and postoperative epilepsy could be influenced by follow-up duration. Third, electroencephalography (EEG) is an important test for diagnosing epilepsy; however, EEG was not performed on all patients, and its data was not included in the analysis. Despite these limitations, this study highlights the occurrence and characteristics of EPSs, DPSs, and de novo epilepsy, and the appropriate usage of ASMs after craniotomy. This study can contribute to help how to treat EPSs and DPSs. Furthermore, this study revealed that postoperative de novo epilepsy was developed about one month after craniotomy, which can be valuable information for surgeons and patients.

The mechanism of de novo epilepsy after craniotomy surgery can be multifactorial, Giraldi suggested that cortical trauma, gliosis, and changes in microcirculation following a craniotomy can be a risk of de novo epilepsy (9). The activation of neuroinflammatory pathways was also reported as a contribution to epilepsy (34). Further basic and clinical studies are necessary to elucidate the mechanism of de novo epilepsy after craniotomy surgery.

## Conclusion

Herein, we reported the clinical characteristics and occurrence of EPSs, DPSs, and de novo epilepsy after craniotomy. Our study revealed that EPSs and DPSs occurred in 4.3% and 4.7% of the patients, respectively. De novo epilepsy occurred in 4.9% of the patients. EPSs and DPSs are risk factors for de novo epilepsy. Previous epilepsy was not a significant risk factor for EPSs. The prophylactic use of ASMs did not prevent EPSs or de novo epilepsy.

## Data Availability

The original contributions presented in the study are included in the article/supplementary material, further inquiries can be directed to the corresponding author/s.
